# Knockdown of CSNK2ß suppresses MDA-MB231 cell growth, induces apoptosis, inhibits migration and invasion

**DOI:** 10.17179/excli2020-2363

**Published:** 2020-09-07

**Authors:** Shibendra Kumar Lal Karna, Bilal Ahmad Lone, Faiz Ahmad, Nerina Shahi, Yuba Raj Pokharel

**Affiliations:** 1 Faculty of Life Science and Biotechnology, South Asian University, Akbar Bhawan, Chanakyapuri, New Delhi-110021, India

**Keywords:** CSNK2beta, breast cancer, apoptosis, migration

## Abstract

Breast cancer is the most common cancer among women worldwide. Among different types of breast cancer known, treatment of triple-negative breast cancer is a major challenge because of its aggressiveness and poor prognosis; thus, identification of specific drivers is required for targeted therapies of breast cancer malignancy. Protein Casein Kinase (CSNK) is a serine/threonine kinase that exists as a tetrameric complex consisting of two catalytic (α and /or α') and two regulatory β subunits. CSNK2β can also function independently without catalytic subunits and exist as a distinct population in cells. This study aims to elucidate the role of Casein Kinase 2β (CSNK2β) gene in cell proliferation, cell cycle, migration and apoptosis of triple-negative breast cancer MDA-MB-231 cells. The silencing of CSNK2β in MDA-MB-231 cells resulted in decreased cell viability and colony formation. Cell cycle analysis showed a significant arrest of cells in G2M phase. Hoechst and CM-H2DCFDA staining showed nuclear condensation and augmented intracellular reactive oxygen species (ROS) production. Furthermore, silencing of CSNK2β in MDA-MB-231 cells modulated the apoptotic machinery- BAX, Bcl-xL, and caspase 3; autophagy machinery-Beclin-1 and LC3-1; and inhibited the vital markers (p-ERK, c-Myc, NF-κB, E2F1, PCNA, p38-α) associated with cell proliferation and DNA replication pathways. In addition, knockdown of CSNK2β also affected the migration potential of MDA-MB-231, as observed in the wound healing and transwell migration assays. Altogether, the study suggests that CSNK2β silencing may offer future therapeutic target in triple-negative breast cancer.

## Introduction

Casein Kinase 2(CSNK2), a highly conserved, multifunctional serine/threonine protein kinase, is critically important for the regulation of different processes in eukaryotes, such as proliferation, differentiation, and apoptosis (Guerra and Issinger, 1999[16]). CSNK2 is ubiquitously expressed in all tissues, but its level is elevated in tumor tissues including the prostate (Yenice et al.,1994[57]), mammary gland (Landesman-Bollag et al., 2001[30]), head and neck (Faust et al.,1996[12]), lungs (Daya-Makin et al., 1994[9]) and kidney (Stalter et al., 1994[50]). CSNK2 possesses a heterotetrameric conformation with two catalytic (α and/or α') and two regulatory (2β) subunits (Litchfield, 2003[34]). CSNK2β supports the structure of the tetrameric complex, augments catalytic activity, the stability of CSNK2, and can also function independently with other catalytic subunits (Litchfield, 2003[34]). In mammalian cells, CSNK2β is phosphorylated at Ser 209 and autophosphorylated at Ser 53B in a cell cycle-dependent manner (Messenger et al., 2002[37]). CSNK2β is responsible for the recruitment of CSNK2 substrates or potential regulators such as Nopp140, p53, Fas-associated factor-1 (FAF-1), topoisomerase II, CD5 and fibroblast growth factor-2 (FGF-2) (Li et al., 1997[32]; Filhol et al., 1992[14]; Appel et al., 1995[3]; Jensen et al., 2001[24]). Ectopic expression of CSNK2β in mouse 3T3-L1 adipocytes and in CHO cells increased proliferation (Li et al., 1999[31]). The proliferative effects of CSNK2β vary in different cell lines. Deletion of gene encoding CSNK2β in mice leads to a failure in development (Ahmed et al., 2002[2]). In our previous work, we have shown CSNK2β among the top 10 Pin1 interacting proteins that might be contributing the oncogenic activity of Pin1 (Pokharel et al., 2015[41]), a cis-trans isomerase, and highly expressed in many cancers (Chen et al., 2018[6]). In the same work, it has been found that siRNA knockdown of CSNK2β potently inhibited the oncogene Pin1 that supports the notion that CSNK2β is vital for cancer pathogenesis. In a patient sample-based study, the positive expression correlation was observed between CSNK2β and XIAP in cholangiocarcinoma patients and CSNK2β was significantly associated with TNM stage (P= 0.036) (Zhou et al., 2014[58]). In a recent study, it has been found that traditional Chinese medicine huaier improves the survival rate of breast cancer patients by modulating the linc00339/mir-4656/CSNK2β pathways (Wang et al., 2019[54]). Together these reports suggest the significance of CSNK2β in cancer progression. The independent existence of CSNK2β without its catalytic subunit has also been reported (Krek et al., 1992[29]; Guerra et al., 1999[17]), that suggests CSNK2β can play roles apart from the regulatory function of CSNK. Thus, the biology of CSNK2β, and its CSNK2α independent function particularly in cancer needs to be further explored.

To understand its independent physiological importance in the regulation of multiple candidate target proteins, we focused our study on the role of CSNK2β in the tumorigenesis of human breast cancer (MDA-MB-231 cell) *in vitro*. In the present study, we used the RNA interference strategy to knockdown the CSNK2β gene and studied the gross oncogenic activity in an* in vitro* cell-based system. We evaluated its proliferative, clonogenic, invasive, and apoptotic properties in MDA-MB-231 cells using siRNA technology. We found that CSNK2β regulates the cell proliferation by targeting NF-κB, Wnt, and MAPK pathway proteins. Our findings suggest that CSNK2β can be used as a novel target for breast cancer therapy.

## Materials and Methods

### Reagents

Lipofectamine RNAiMAX, TRIzol, Propidium Iodide, RNase were purchased from Invitrogen Corp (Carlsbad, CA, USA). siRNA was obtained from Qiagen (Hilden, Germany). Cell culture reagents and flasks were purchased from HiMedia (France) and Corning Inc. (Corning, New York, USA). SYBR Green was purchased from Bio-Rad (Hercules, California). Antibodies were obtained from Santa Cruz Biotechnology (Dallas, Texas, USA), Cloud-Clone Corp. (Houston, USA). Cell Titer-Glo reagent was purchased from Promega Corp. (Madison, Wisconsin, USA).

### Cell culture

MDA-MB-231 cell was purchased from National Centre for Cell Science (Pune, India). The cells were cultured in L-15 medium supplemented with 10 % Fetal Bovine Serum (FBS), penicillin (100 unit/ml) and streptomycin (100 µg/ml). The cell culture was maintained at 37 °C in humidified air containing 5 % CO_2_.

### Transfection

We used functionally verified siRNA directed against human CSNK2β (NM_001320), purchased from Qiagen with no potential off-targets prevalidated in HeLa cell line (Catalog Number: SI00605185). Also, predesigned negative control siRNA (Scramble) from Qiagen was used in our experiments (Catalog Number /ID: 1027310). Cells were cultured in 6 well plates one day before siRNA transfection. We used 25 nanomolar of each siRNA and made complex in Opti-MEM media. Similarly, the complex of Lipofectamine RNAiMAX (4 µl/each well) and Opti-MEM was made and incubated for 5 minutes at room temperature. After that, both the complexes were mixed in 1:1 proportion and incubated for 25 minutes at room temperature. Cells were treated with Opti-MEM-siRNA-Lipofectamine complex and incubated at 37 °C for 72 hours. The sequences used in siRNA and the target sequence for the genes in our study are mentioned below (Table 1(Tab. 1)).

### Cell viability assay

Cell viability was assessed with CellTiter-Glo (CTG) assay (Promega, Madison, WI). Briefly, MDA-MB-231 cells were seeded in 96 well white cell culture plate at a density of 3000 cells per 180 µl of medium per well with 20 µl of siRNA complexes for CSNK2β and Scramble and incubated at 37 °C, 5 % CO_2 _for 24 hours. On the next day, the media containing the complex was changed with the fresh media and further incubated till 96 hours. The cells were treated in quadruplets with respective siRNAs. The reagents were prepared according to the manufacturer's protocol. After incubation, 100 µl of fresh media was added to each well followed by 100 µl of reagent and kept on a shaker for 2 minutes to induce the cell lysis. The plate was incubated for 10 minutes at room temperature to stabilize the luminescence signal. Luminescence was measured using a microplate ELISA reader (Bio Tek, Winooski, Vermont, US).

### Colony formation assay

MDA-MB-231 cells were transfected with Scramble and CSNK2β siRNA and incubated for 48 hours. After the cells were trypsinized, collected and counted, 500 cells/well were taken from each Scramble and CSNK2β transfected samples and seeded in 6 wells plate. After every two days, the medium was changed, and the cells were grown for three weeks. Then the cells were washed with Dulbecco's Phosphate Buffered Saline (DPBS), fixed with 3.7 % formaldehyde for 10 min and stained with 0.4 % crystal violet. The cells were washed with DPBS for 2-3 times and allowed to dry. The colonies were counted using Image J software.

### Wound healing assay

MDA-MB-231 cells were plated in 6 well plates (4×10^5^ cells/well) and transfected with Scramble and CSNK2β siRNA as mentioned above. After the cells reached 90 % confluence, a wound was made in the center of the plate by scrapping the cells monolayer with 10µl tip. The cells were washed with sterile DPBS to remove the detached cells. The width of the wound area was measured at different time points using an inverted microscope. Densitometry analysis of wound area was carried by using Image J software.

### Matrigel invasion assay

Transwell chambers (Corning) were used for invasion assay. MDA-MB-231cells were transfected with CSNK2β siRNA and post 48 hours of transfection, cells (5 X 10^4^) were harvested and transferred to the upper chamber of transwell, pre-coated with matrigel, in 200 µL of serum free L-15 media whereas lower chamber consisted of complete media serving as a chemoattractant for the cells. Following 24 hours of incubation, cells in the upper surface of chambers were removed with a cotton swab, and the cells invaded through matrigel at the lower surface were fixed in 70 % ethanol and stained with 0.4 % crystal violet. Quantification of invaded cells was done by counting in six random fields.

### Cell cycle analysis

2.5x10^5^ cells were seeded in 6 well plates and transfection was done as mentioned before. The cell cycle analysis was performed 48 hours post-transfection using BD FACS Verse ™. The cells were pooled out and fixed with ice-cold 70 % ethanol and kept overnight at 4 °C. On the next day, the cells were washed with DPBS for two times. The cells were further digested with RNase A (50 U/ml) at 37 °C for 1 hour and then stained with propidium iodide (20 μg/ml) at room temperature for 20 min. The samples were acquired by flow cytometer and results were analyzed by ModFit software.

### Western blotting

MDA-MB-231 cells were cultured in 6 well plates. On the next day, cells were transfected with Scramble and CSNK2β siRNA and incubated for 72 hours. After incubation, the cells were washed twice with ice-cold DPBS and then lysed with sodium dodecyl sulfate (SDS) lysis buffer. The samples were heated for 5 minutes at 100 °C. The samples were run on SDS-PAGE, transferred to polyvinylidene (PVDF) membrane (90 volts for 2 hours). The membranes were blocked using 5 % skim milk in Tris-buffered saline with 0.1 % tween-20 (TBST) for 2 hours at room temperature. The blots were incubated with primary antibodies overnight at 4 °C on a rocker followed by incubation with horseradish peroxidase (HRP) conjugated secondary antibodies. The blots were developed using enhanced chemiluminescence (ECL, Biorad). β actin was used as a loading control. The list of antibodies with their working dilution is mentioned in Table 2(Tab. 2). 

### RNA extraction, cDNA preparation, and real time PCR 

Cells were transfected for 72 hours with the respective siRNAs and RNA was extracted using TRIzol lysis reagent using manufacturer's protocol. 2 µg of RNA was taken and pre-treated with DNase followed by cDNA synthesis with oligoDT primers. The thermocycler condition was 65 °C, 5 min, 42 °C, 60 min and 72 °C (final extension). Real time PCR was done with iTaq SYBRgreen mix (Biorad) using ABI 700 (Invitrogen). The Real time PCR thermocycler condition was as Hold stage 95 °C (10 min), PCR Stage (40 cycles) 95 °C (15 sec), 60 °C (1 min) and melt curve stage 95 °C (15 sec), 60 °C (1 min), 95 °C (15 sec). Gapdh was used as a housekeeping gene. mRNA expression analysis was done by the delta-delta-Ct method. The expression of each gene in CSNK2β siRNA treated sample was compared with Scramble. The sequences of primers used are mentioned in Table 3(Tab. 3).

### Hoechst staining

1.5x10^5 ^cells were seeded in 6 well plates. On the next day, the cells were transfected with Scramble and CSNK2β siRNA and incubated for 72 hours. Then the cells were stained with Hoechst 33342 for 15 minutes in the dark. Cells were once washed with DPBS and the images were taken using Nikon fluorescent microscope.

### Apoptosis assay 

Cell apoptosis assay was performed by flow cytometry using an Annexin V-FITC/ propidium iodide (PI) apoptosis detection kit (BD Biosciences). Cells were seeded in triplicates in a 6-well plate at the density of 2x10^5 ^cells per well. After 48 hours of transfection with Scramble and CSNK2β siRNA, the cells were harvested and the Annexin V-FITC/PI assay was performed according to the manufacturer's instruction. A total of 30,000 cells were analyzed using FACSuite software. The percentage of cells in different phases of apoptosis was examined.

### Measurement of the intracellular reactive oxygen species (ROS) production

Intracellular reactive oxygen species generation was assessed by fluoroprobe CM-H2DCFDA (Invitrogen). 1.5x10^5^ cells were cultured in 6 well plates and transfected with the Scramble and CSNK2β siRNA for 72 hours. The cells were treated with 10 µM CM-H2DCFDA for 30 min in the dark at 37 °C. The wells were once washed with DPBS and the images were captured using a fluorescence microscope (Nikon Ti).

### Statistical analysis

Data were represented as a mean ± standard deviation. The level of significance between the two groups was calculated by Student's t-test. P < 0.05 was considered statistically significant.

### Bioinformatics 

Mutational status of CSNK2β across different cancers was carried out using cBioPortal (https://www.cbioportal.org/). Kaplan-Meier curves of relapse-free survival for breast cancer patients were evaluated by Kaplan-Meier plotter (KM-potter, https://kmplot.com/analysis/). Cancer and normal gene expression profiling of CSNK2β were examined by GEPIA (http://gepia.cancer-pku.cn/) and MERAV (http://merav.wi.mit.edu/) online servers.

## Results

### Comparative analysis of CSNK2β expression in multiple cancer types and the correlation with overall survival

**Figure 1:**
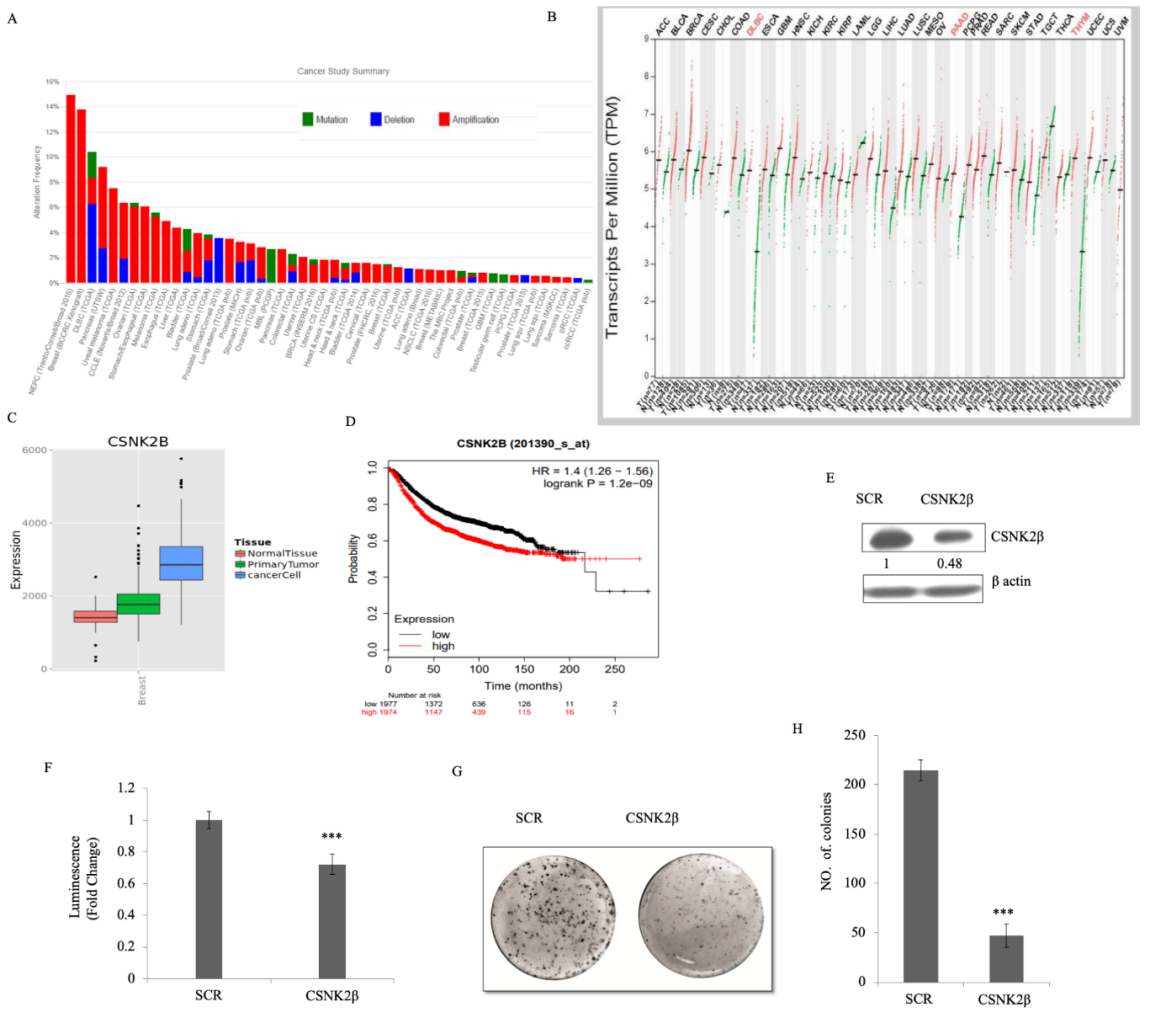
CSNK2β expression in multiple cancer types and its functional role in breast cancer cell proliferation. (A) Mutational status of CSNK2β across human cancers based on cBioPortal data. Histogram representing the mutational landscape (mutation, amplification, and deep deletion) of CSNK2β in different types of cancer. (B) The gene expression profile across all tumor samples and paired normal tissues using GEPIA database. For each tumor (red), its matched normal (green) are given; T: tumor; N: normal; n: number. Y-axis represents a transcript per million in log scale, whereas X-axis represents the number of tumors and normal samples. Each dot represents expression of samples; the data for breast cancer lie in a third column. (C) Comparison of gene expression between breast tumor tissue and adjacent normal tissue using MERAV database. (D) Kaplan-Meier survival curve of the mRNA expression of CSNK2β for breast cancer patients (n=3951) using the KM-plotter database. (E and F) Silencing of CSNK2β and evaluation of its expression by western blot followed by determination of cell viability by CTG assay. The values below the blot represent the densitometry of the corresponding band compared to loading control β actin. The experiments were performed in triplicates. (G and H) The knockdown of CSNK2β decreases the colony formation potential of MDA-MB-231 cells. Bar graphs depict the number of colonies in each sample. Data are represented as mean ± SD from triplicate samples, where *** denotes p < 0.001.

To understand the expression status of CSNK2β in different cancers, we used the datasets retrieved from cbioportal (Cerami et al., 2012[5]) and compared the genomic alterations in multiple tumors. CSNK2β showed a high degree of amplification in most cancer types followed by deletion and mutation (Figure 1A(Fig. 1)). The TCGA data retrieved from cbioportal shows about 14 % gene amplification of CSN2β in breast cancer. Next, we checked the expression status of CSNK2β in different cancers using the GEPIA database (Tang et al., 2017[52]). Among 32 different cancers showed in the panel, expression is significantly higher in bladder urothelial carcinoma, breast invasive carcinoma and mesothelioma (Figure 1B(Fig. 1)). We also observed the distribution of CSNK2β gene expression between breast tumor tissue and adjacent tissue using MERAV database (http://merav.wi.mit.edu/) (Shaul et al., 2016[46]). As shown in Figure 1C(Fig. 1), the expression of CSNK2β is upregulated in breast tumors. To evaluate whether CSNK2β could be a prognostic factor for breast cancer patients, we examined mRNA expression status of CSNK2β in a large cohort of datasets containing 3951 breast cancer patients with online KM-Plotter software (http://kmplot.com/analysis) (Györffy et al., 2010[20]) and analyzed the correlation between CSNK2β expression and patient survival. Kaplan-Meier analysis using KM-plotter revealed that a group with higher CSNK2β expression have significantly poorer relapse-free survival (RFS) (p<0.001, longrank) (Figure 1D(Fig. 1)). The correlation of CSNK2β gene expression and RFS was evaluated using microarray data set containing breast cancer expression profiling from Gene Expression Omnibus (GEO). Kaplan-Meier estimates of RFS were calculated by splitting the patients into high- and low-expressing groups. The hazard ratio, log-rank *P*-value and number of breast cancer patients in each group are shown on the KM plot. Together, these results suggest that CSNK2β is clinically important for cancer pathogenesis and correlates with patient outcome.

### Silencing of CSNK2β inhibited the proliferation of MDA-MB-231cells

To study the knocking down effect of CSNK2β *in vitro*, we silenced the expression of CSNK2β using the siRNA approach and evaluated the CSNK2β protein level. The result showed the silencing of CSNK2β was efficient compared to scrambled siRNA (SCR) (Figure 1E(Fig. 1)), Next, cell viability was measured in 96 hours of post-transfection by using CTG substrate. The viability of CSNK2β silenced MDA-MB-231 cells was significantly lower as compared to Scramble siRNA (p< 0.001) (Figure 1F(Fig. 1)). These results suggested that CSNK2β play a crucial role in cell proliferation of MDA-MB-231 cells. Clonogenic assay which mimics the tumor formation *in vivo* was carried out to observe the long-term survival of cells, which were transfected with CSNK2β +/- siRNAs. Silencing of CSNK2β significantly reduced the number of colonies as compared to Scramble siRNA transfected cells (p< 0.001) (Figure 1G, H(Fig. 1)). This result further confirmed the pivotal role of CSNK2β in the proliferation of MDA-MB-231 cells.

### Silencing of CSNK2β augmented intra-cellular reactive oxygen species (ROS)production, caused cell cycle arrest at G2/M and induced apoptosis in MDA-MB-231 cells in vitro

**Figure 2:**
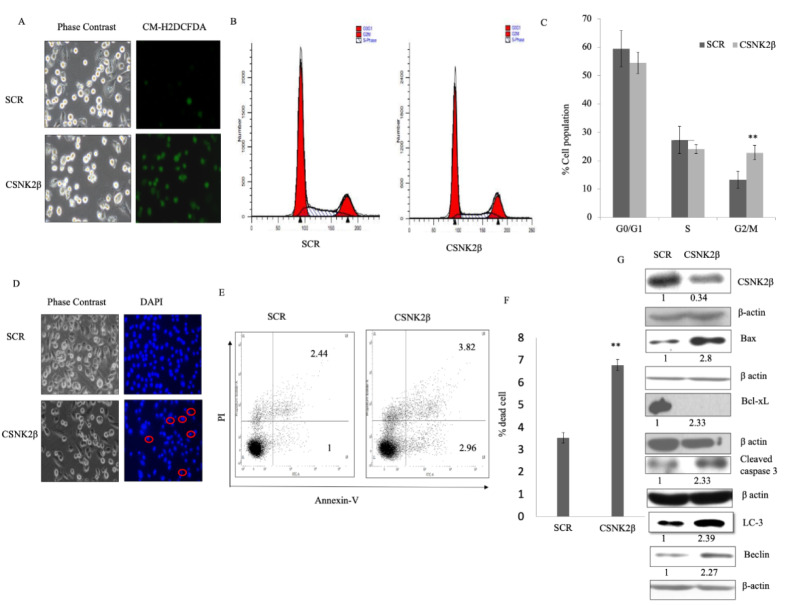
Silencing of CSNK2β induced apoptosis in MDA-MB-231 cells *in vitro.* (A) Representative fluorescent microscopy image of Intracellular ROS production using CM-H2DCFDA dye in Scramble and CSNK2β siRNA treated cells. (B and C) Representative images of cell cycle analysis after treating the cells with Scramble and CSNK2β siRNA for 48 hours, represented as histogram and bar graph. (D) Representative images of microscopic evaluation of cell morphology and Hoechst stained nuclei after siRNAs transfection. Condensed nuclei of apoptotic cells are circled as characterized by a smaller and fragmented appearance with respect to control. (E and F) Identification of Apoptosis with Annexin V-FITC/PI double staining by flow cytometry. Distribution of cells in different phases of apoptosis are represented as dot plot diagram in following manner. Lower left: viable cells; Lower right: early apoptotic cells; Upper left: Necrotic cells; and Upper right: late apoptotic cells. Bar graph representation shows total apoptotic cells in both siRNAs treated cells (early and late apoptosis). (G) Representative image of western blot analysis of apoptosis associated markers. Knockdown of CSNK2β inhibited Bcl-xL whereas activated Bax, caspase 3, Beclin-1 and LC-3. The values below the blot represent the densitometry of the corresponding band compared to loading control β actin. The experiments were performed in triplicates. Results for Annexin V-FITC/PI double staining are representative of two independent experiments represented as a mean ± standard deviation, where **p < 0.01.

ROS possesses double edge sword property both as, oncogenic- maintaining sustained and increased proliferation of cancer cells, as well as, tumor suppressor- leading to cell death when an aberrant increase in intracellular ROS arises due to any kind of stress. It is a good idea to find oxidative stress modulators as an anti-cancer strategy (Wang and Yi, 2008[53]; Gurer-Orhan et al., 2018[19]). In our experiment, we found the increased production of ROS in CSNK2β siRNA transfected cells compared to scramble siRNA transfected cells (Figure 2A(Fig. 2)). This data suggested that CSNK2β plays a crucial role in attenuating the intracellular ROS production in MDA-MB-231 cells for its sustained proliferation. 

Next, we checked the status of the cell cycle phase following knockdown of CSNK2β. After 48 hours of transfection, the percentage of cells in the different phases of the cell cycle were analyzed by flow cytometry. The percentage of cells in G2/M phase was significantly increased in CSNK2β transfected samples (22.806 %) than in Scramble siRNA transfected samples (13.266 %) (p < 0.01). At the same time, the percentage of cells in G0/G1 and S phase was reduced (Figure 2 B, C(Fig. 2)). These results suggest that knockdown of cells with CSNK2β inhibits the progression of cells via blocking the cell cycle at G2/M phase. 

The significant arrest of MDA-MB-231 cells in G2/M phase led us to check the effect of CSNK2β knockdown on cell apoptosis. We found that silencing of CSNK2β caused change in cellular morphology and condensation of nuclei - a prominent feature of cells undergoing apoptosis (Figure 2D(Fig. 2)). Quantitative estimation of apoptotic cells was carried out using flow cytometry with annexin V-FITC and PI. Our data showed 6.77 % of apoptosis (early apoptotic + late apoptotic population) in CSNK2β siRNA knockdown cells compared to 3.52 % (early apoptotic + late apoptotic population) in Scramble siRNA cells which comes out to be significant with p < 0.01 as shown in (Figure 2E, F(Fig. 2)). To unravel the cell death mechanism induced after the knockdown of CSNK2β, western blot analysis was performed to determine the level of proteins related to apoptosis. We found an increased expression of BAX and cleaved caspase 3 along with a decreased level of Bcl-xL expression (Figure 2G(Fig. 2)). Altogether these results showed that the silencing of CSNK2β drives cell death via apoptosis. Moreover, we also analyzed the expression of two vital autophagy markers, Beclin-1 and LC3, and found both markers were upregulated in CSNK2β siRNA transfected samples than Scramble siRNA (Figure 2G(Fig. 2)). These findings together showed that targeting CSNK2β triggers the cells towards both apoptosis and autophagy cell death pathway.

### Knockdown of CSNK2β inhibited migration and invasion of MDA-MB-231 cells

**Figure 3:**
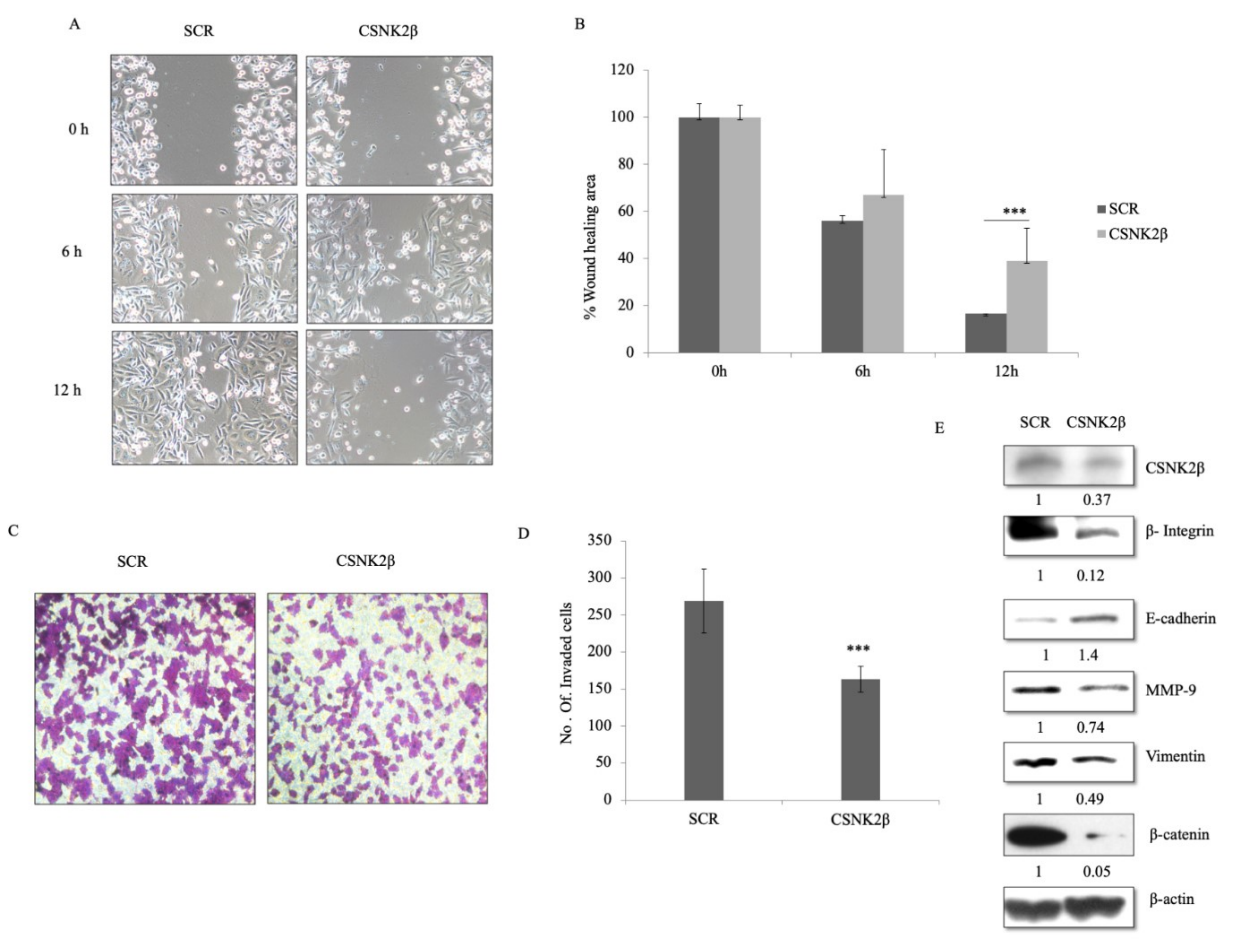
Silencing of CSNK2β inhibited migration and invasion of MDA-MB-231 cells. (A and B) Representative images of wound healing assay performed to evaluate the motility of cells after silencing CSNK2β. After transfection, a scratch was made on cells monolayer and was monitored with microscopy every 6 hours (0, 6, and12 h). Bar graphs show normalized wound area, calculated using Image J software. (C and D) Representative images of invasion assay. Bar graphs depict number of invaded cells following knockdown of CSNK2β. Data are represented as mean ± SD from triplicate samples, where **p <0.01 and ***p < 0.001. (E) Representative image of western blot analysis depicts protein expression of migration associated markers. Knockdown of CSNK2β upregulated E-cadherin whereas downregulated β integrin1, MMP-9, and β-catenin. The values below the blot represent the densitometry of the corresponding band compared to loading control β actin. The experiments were performed in triplicates.

Next, we questioned whether the depletion of CSNK2β could alter the migration and invasion potential of MDA-MB-231 cells. In wound healing assay, migratory capacity was observed for each sample at different time points (0, 6, and 12 h). After 12 hours, the area of a wound was significantly wide in CSNK2β transfected wells as compared with Scramble suggesting the role of CSNK2β gene in the migration of MDA-MB-231 cells *in vitro* (p < 0.001) (Figure 3A, B(Fig. 3)). Next, we also checked the invasive potential of MDA-MB-231 following CSNK2β knockdown by transwell invasion assay. We found that cells transfected with CSNK2β siRNA were unable to invade the matrigel layer as efficient as the Scramble siRNA transfected cells (Figure 3C, D(Fig. 3)). Some critical markers associated with cell migration were also checked by western blotting and it was found that knockdown of CSNK2β caused inhibition of MMP-9, Vimentin, β Integrin1, and β-catenin along with upregulation of E-cadherin (Figure 3E(Fig. 3)).

### Silencing of CSNK2ß modulated key markers associated with cell proliferation, migration, and cell stemness

**Figure 4:**
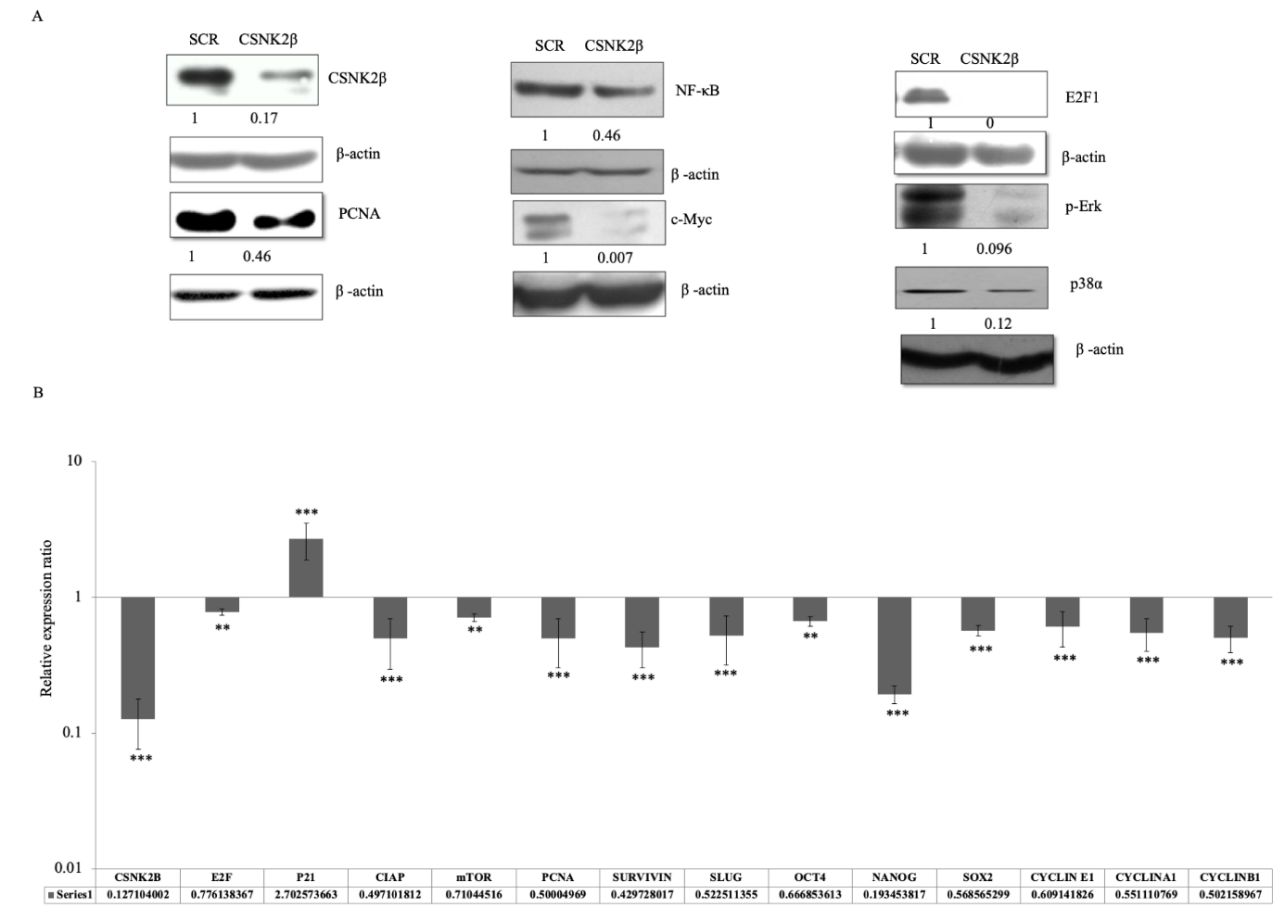
Silencing of CSNK2β modulated key markers associated with cell proliferation, migration, and cell stemness. (A) Representative image of the western blot analysis of proteins associated with cell proliferation. The values below the blot represent the densitometry of the corresponding band compared to loading control β actin. The experiments were performed in triplicates. (B) qPCR analysis of genes associated with apoptosis, cell cycle, cell stemness, and metastasis. Data are represented as mean ± SD from triplicate samples, where **p <0.01 and ***p < 0.001.

To elucidate the role of CSNK2β in MDA-MB-231 cell proliferation, survival, and apoptosis, we examined the expression of some selected genes related to these phenomena at mRNA and protein level by real time PCR and western blotting respectively. Knockdown of CSNK2β led to downregulation of PCNA, E2F1, c-Myc, NF-κB, p-ERK, and p-38α protein level, as shown in (Figure 4A(Fig. 4)). In 72 hours transfected samples, we found that the mRNA level of p21, a cell cycle inhibitor, was increased up to about 2.7 fold, while the expressions of survivin (anti-apoptotic and metastatic marker), Nanog, SLUG, OCT4, SOX2 (transcription factor for stemness) and cyclin E1, Cyclin A1, cyclin B1 (cell cycle cyclins) and E2F1, mTOR, PCNA proliferative marker gene expression were decreased following the knockdown of CSNK2β (Figure 4B(Fig. 4)). These results showed the critical role of CSNK2β in the expression of genes associated with cell cycle, cancer stemness, and metastasis. 

## Discussion

Breast cancer is the most commonly diagnosed as well as the leading cause of cancer deaths in women (Bray et al., 2018[4]). Mortality due to breast cancer has been decreasing due to early diagnosis, improved adjuvant therapy, and low usage of hormone replacement therapy (Ravdin et al., 2007[43]). In our study, we chose to elucidate the role of CSNK2β in breast cancer (MDA-MB-231, a TNBC cell line) *in vitro*.

CSNK2 is a serine/threonine kinase that exists in a heterotetrameric complex consisting of two catalytic (α and/or α') and two regulatory (2β) subunits. CSNK2β exists and functions either in a complex or independent population in cell. Here we report the independent function of CSNK2β in MDA-MB-231 cells. Our results showed that knockdown of CSNK2β significantly decreased the cell viability and colony growth of MDA-MB-231 cells. Based on these observations, we next evaluated the status of intracellular reactive oxygen species (ROS) inside the MDA-MB-231 cells, since the controlled production of ROS is beneficial for proliferation and viability of cancer cells but a disproportional increase of ROS can induce cell cycle arrest and at its peak can induce apoptosis of cancer cells (Gurer-Orhan et al., 2018[19]; Liou and Storz, 2010[33]). In our study, we found that knockdown of CSNK2β markedly induced ROS production which seems to be consistent with earlier finding that use of a pharmacological inhibitor which blocks the catalytic activity of CSNK2 induced apoptosis in leukemic cell lines via up-regulation of intracellular H_2_O_2_ (Hanif et al., 2009[21]). Moreover, we found the G2M cell cycle arrest of MDA-MB-231 cells following knockdown of CSNK2β, and in some studies, it has been shown that ROS accumulation is at its peak in the G2M phase of cell cycle (Havens et al., 2006[22]). So the accumulated ROS found in this study can be attributed to arrest of cells at G2/M phase following knockdown of CSNK2β. However, the exact mechanism which triggered redox modulation upon knockdown of CSNK2β was not explored in this study and further investigation is needed to understand the association of CSNK2β and the ROS status inside MDA-MB-231 cancer cells. 

A perturbed cell cycle progression may lead to apoptosis (Pucci et al., 2000[42]). In our study, we found siRNA knockdown of CSNK2β in MDA-MB-231 led to nuclear condensation and change of cellular morphology, a characteristic of cell undergoing apoptosis. Further, this study also showed that depletion of CSNK2β modulated molecular markers associated with apoptosis and autophagy, which is consistent with earlier reports of involvement of CSNK2 in suppressing apoptosis, as overexpression of CSNK2 in prostate cancer cells prior to treatment with etoposide rescued against cell death (Guo et al., 2001[18]) and downregulation of CSNK2 (α subunit) leads to autophagic cell death in glioblastoma cell lines (Olsen et al., 2012[38]). These results suggest that CSNK2β has a prominent role in the survival of MDA-MB-231 cells by suppressing the apoptotic and autophagy machinery.

In this study, we also explored the role of CSNK2β in metastasis and invasion, as these two important phenotypes are associated with breast cancer. Metastasis, which is evident from stage 2 onwards and nearly 30 % of women develop metastasis even after initial diagnosis (O'Shaughnessy, 2005[39]), requires identification of markers involved in this lethal property of cancer. In the present study, we found that silencing of CSNK2β markedly up-regulated E-cadherin that hinders invasiveness in carcinomas and other cancers (Singhai et al., 2011[49]). In search of other markers associated with migration and hence metastasis, being affected following inhibition of CSNK2β, we found down-regulation of β1 Integrin, MMP-9, Vimentin, and β-catenin. β1 Integrin was found to be inhibited in CSNK2β knockdown cells, which is a majorly expressed integrin and has been reported to actively be involved in metastasis and has attracted considerable attention as a target for immunotherapy in breast cancer (Park et al., 2008[40]). MMP-9, a protease, facilitates cancer cells in degrading extracellular matrix for metastasis and invasion, has been found to be overexpressed in many cancers, including breast cancer. We found MMP-9 to be inhibited following knockdown of CSNK2β, which corroborates with the previous finding of MMP-9 inhibition with chemically inhibited CSNK2 in lung adenocarcinoma cells (Kim and Kim, 2013[28]). Vimentin, a major factor associated with epithelial-mesenchymal transition (EMT) (Satelli and Li, 2011[44]) and a vital prognostic biomarker and therapeutic target correlated with EMT in TNBC (Yamashita et al., 2013[56]) was found to be downregulated in CSNK2β knockdown cell. In addition to these, we also found that knockdown of CSNK2β downregulated the expression of β-catenin. It has been previously shown that β-catenin plays a pivotal role in tumor-related phenotypes, including migration and stemness, in particular in TNBC cells (Xu et al., 2015[55]). Collectively, these results showed that CSNK2β mediates migration and invasion of MDA-MB-231cells, and these critical markers appear to be acting downstream of CSNK2β. 

To get further insight into the biological significance of CSNK2β in the pathogenesis of breast cancer, we performed the expression study of the key molecules related to cell proliferation, survival, cell cycle, apoptosis, and autophagy-related genes by western blotting and real time PCR.

Raf/MEK/ERK pathways are activated in many tumors (prostate, breast, leukemia, melanoma, thyroid), which transmit the signals from cell surface receptors to transcription factors and can be exploited for therapeutic intervention (McCubrey et al., 2007[36]). Here, we found that CSNK2β regulates the expression of p-ERK, p38-α, c-Myc proteins, which are connected to the MAPK pathway in MDA-MB-231 cells. These data suggested that targeting CSNK2β might be a potential strategy to improve clinical outcomes in the future. PCNA has been earlier reported as a reliable marker to access the growth and predicting the prognosis in breast cancer (Schönborn et al., 1994[45]). Thus, we investigated the effect of silencing of CSNK2β on PCNA and found its downregulation at both transcript and protein levels. A recent study on the interbreeding of MMTV-PyMT mice with E2F1, E2F2, or E2F3 knockout mice showed that in addition to cell cycle control E2F targets a number of genes related to angiogenesis, extracellular matrix modification, proliferation and survival of tumor cells which was important for metastasis (Hollern et al., 2014[23]). We also found that CSNK2β reduces the expression of E2F1, which might affect a large fraction of genes in breast cancer. NF-κB expression leads to the induction of genes related to apoptosis, cell cycle, cell invasion which contributes to tumorigenesis, chemoresistance, and radioresistance (Greten and Karin, 2004[15]). Our result showed that CSNK2β might regulate the cell proliferation through the NF-κB pathway. 

One of the most striking results obtained in this study following silencing of CSNK2β was a highly significant upregulation of p21 at the transcript level. p21 is well known as sensor and effector of multiple growth inhibiting signals and its overexpression has been reported to cause arrest in breast carcinoma cell lines (Sheikh et al., 1995[47]). This result is in a similar line of an earlier report where inhibition of CSNK2 using inhibitor caused up-regulation of p21 in glioblastoma cells and induced G2M arrest of cell cycle (Dixit et al., 2012[10]). Knockdown of CSNK2β also led to the downregulation of mTOR. Alteration in mTOR pathway is a common anomaly in various breast cancer subtypes (Costa et al., 2018[8]). We also checked the expression of survivin and cIAP as both are inhibitors of apoptosis and overexpressed in the majority of breast cancer; moreover, survivin has been found to confer resistance to chemotherapy (Jha et al., 2012[25]). In this study, we also found that expressions of slug, oct4, nanog and sox2 were significantly downregulated at transcript level. Slug overexpression has been reported to induce stemness and promote migration in hepatocellular carcinoma, breast and other cancer types (Sun et al., 2014[51]; Ferrari-Amorotti et al., 2014[13]), and its overexpression is associated with reduced expression of E-cadherin in lymph node metastasis and poor survival of patients (Shioiri et al., 2006[48]). Interestingly, we found that knockdown of CSNK2β led to downregulation of slug and upregulation of E-cadherin. Sox2, a transcription factor, plays an important role in the pluripotency of stem cells. Downregulation of Sox2 is a promising outcome of this study as Sox2 has been reported to positively influence cell proliferation and migration of TNBC cells (Liu et al., 2018[35]). Downregulation of oct-4 and nanog is another crucial finding of this study since both of the pluripotency factors are highly expressed in multiple cancers (Ezeh et al., 2005[11]; Chiou et al., 2010[7]) including various subtypes of breast cancer and their abrogation reverses epithelial-mesenchymal transition in lung carcinoma (Chiou et al., 2010[7]). Although this is a very initial finding for the relationship between CSNK2β and mentioned stemness marker, a further study is needed to explore the signal relationship among them.

We also explored the expression level of cyclins (A1, B1, and E1) at the transcript level, following knockdown of CSNK2β. These cyclins have been shown to be associated with breast cancer progression, via modulating different cellular pathways such as by modulating VEGF and hence angiogenesis (Khaja et al., 2013[27]) and poor survival of patients (Aaltonen et al., 2009[1]; Keyomarsi et al., 2002[26]).

In conclusion, the present study explored the independent function of β subunit of CSNK2 in breast cancer pathogenesis in MDA-MB-231 cells. Our study has shown that silencing of CSNK2β significantly inhibited the growth of MDA-MB-231 cells, caused cell cycle arrest, and induced apoptosis. Further, this study also found that CSNK2β knockdown significantly hampered migration and invasion of MDA-MB-231 cells via downregulation of MMP-9, vimentin, beta-integrin 1, and up-regulation of E-cadherin. Our study has paved the way for further investigation to establish the role of β subunit in different cancers and diseases. 

## Authors’ contribution

Shibendra Kumar Lal Karna (SKLK) executed most of the experiments and wrote the manuscript, Bilal Ahmad Lone (BAL), performed some experiments and manuscript writing, Faiz Ahmad (FA) contributed some experiments and manuscript writing, Nerina Shahi (NS) performed part of the experiments, Yuba Raj Pokharel (YRP) designed, supervised and edited the manuscript.

## Conflict of interest

The authors declare that they have no competing interests.

## Funding

SAU-START-UP-GRANT-2014, South Asian University, New Delhi, India. Funding sources had no role in the design of the study, analysis of data, and writing the manuscript.

## Figures and Tables

**Table 1:**
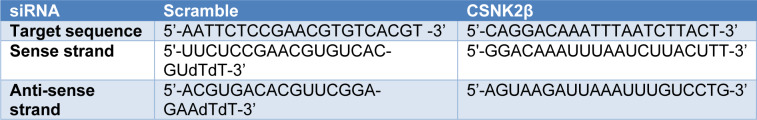
Sequences of siRNAs used for transfection of cells

**Table 2:**
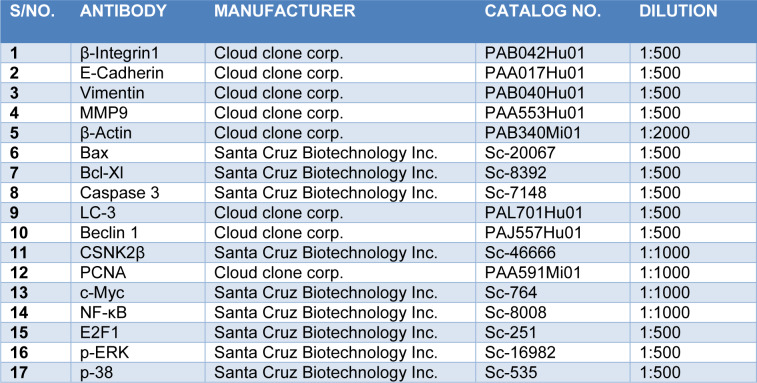
List of antibodies used for western blot analysis

**Table 3:**
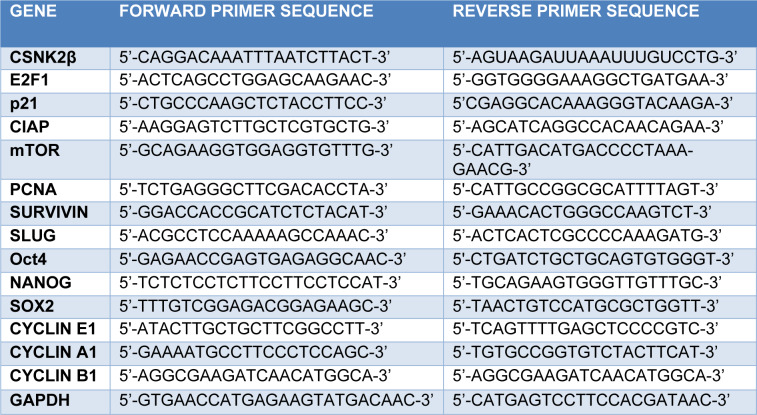
Sequences of primers used for mRNA expression analysis by Real time PCR
